# Adversity Tries Friends: A Multilevel Analysis of Corporate Philanthropic Response to the Local Spread of COVID-19 in China

**DOI:** 10.1007/s10551-021-04745-z

**Published:** 2021-02-17

**Authors:** Hanwen Chen, Siyi Liu, Xin Liu, Daoguang Yang

**Affiliations:** 1 School of Government Auditing, Nanjing Audit University, 86 West Yushan Road, Nanjing, 211815 P.R. China; 2 UIBE Business School, University of International Business and Economics, No.10 Huixin Dongjie, Beijing, 100029 P.R. China

**Keywords:** Corporate philanthropy, COVID-19, Economic uncertainty, Strategic motivation

## Abstract

We examine corporate philanthropic decisions in response to the local spread of COVID-19. From a strategic perspective, firms may proactively undertake philanthropic efforts to limit the spread of the pandemic and avoid a degraded business environment. From the perspective of non-trivial costs, increased economic uncertainty can raise concerns about business survival and lead to conservative philanthropic strategies. Following the proverb “prosperity makes friends, adversity tries them,” at the provincial level, our results support the second perspective. Specifically, when the spread of the pandemic worsens in a province, local firms are less likely to make COVID-19-related donations in terms of likelihood and amount. Investors also react negatively, not only to the local spread of COVID-19 but also to COVID-19-related philanthropic donations. At the organizational level, our evidence indicates that there is at least some level of cost–benefit analysis underlying corporate philanthropic decisions. Specifically, corporate philanthropic donations, especially those made to the local business environment, are significantly affected by organizational-level factors, such as pre-existing resource availability and motives to acquire political and reputational resources. Overall, our multilevel study presents a comprehensive picture of corporate philanthropic decisions amid the COVID-19 crisis.

## Introduction

The 2019 novel coronavirus outbreak (COVID-19 hereinafter) has led not only to a public health crisis but also to a global economic crisis (World Bank 2020). While governments should bear primary responsibility for limiting the spread of this pandemic and mitigating its influence on the economy, a remarkable trend has been the outpouring of corporate philanthropic donations in reaction to the unprecedented shortage of medical supplies (e.g., masks and antiviral drugs), especially at the early stage of the outbreak. Although this type of effort can help control the pandemic, in general, philanthropy is seen as a “nice-to-do” rather than a “must-do.” In the pyramid model of corporate social responsibility (CSR hereafter), Carroll (1991) depicts the role of philanthropy as “icing on the cake—or on the pyramid” (p. 42). Hence, it makes sense that during difficult times, firms striving for economic gains should be reluctant to proactively allocate their corporate resources to tackle social problems. One recent example of such reluctance is that of the Chinese listed firm Zhejiang Juli Culture Development Corporation, when a COVID-19 donation proposal sparked strong discontent from one director (SINA Finance 2020). According to this director, this donation was not affordable for a firm that has suffered losses for two consecutive years and whose employee wages have been in arrears for months. This type of anecdote inspires us to investigate firms’ philanthropic decisions in response to the spread of COVID-19 in their local business environment.

There are still ambiguities regarding the definition of corporate philanthropy (Gautier and Pache 2015; Liket and Simaens 2015). In general, corporate philanthropy can “involve donating money, products or services, as well as volunteering” (Liket and Simaens 2015, p. 285). During the pandemic, virus transmission is expected to severely restrict volunteer activities (Fidelity Charitable 2020). Besides, there is no clear distinction between many corporate volunteering activities and operating activities.^1^ Hence, we follow prior studies (e.g., Muller and Kräussl 2011b) and strictly define corporate philanthropy as donations of cash and supplies.

The COVID-19 crisis provides an ideal setting to explore the influence of local environmental changes on corporate philanthropy, thereby addressing the criticism of the ignorance of “institutional dynamics” (Gautier and Pache 2015, p. 362) in the literature. Based on the literature on strategic philanthropy, corporate philanthropy has the potential to improve not only social warfare but also long-term benefits to firms, leading to a win–win outcome (Post and Waddock 1995; Saiia et al. 2003; Maas and Liket 2011). As the quality of the business environment is important for a firm’s competitive advantage, corporate philanthropy can be viewed as an investment to improve the business environment even though it represents an outflow of economic resources (Porter and Kramer 2002; Gautier and Pache 2015). From this perspective, when the local business environment deteriorates, firms have an incentive to proactively allocate corporate resources to limit the spread of COVID-19 and to reduce the economic uncertainty induced by the pandemic.^2^ While this strategic perspective predicts a positive corporate philanthropic response to the local spread of COVID-19, it may have underestimated the effect of the non-trivial costs imposed by adverse events (Godfrey et al. 2009). Specifically, when uncertainty in the local environment becomes too high, firms may switch to defensive approaches to social issues (Pondeville et al. 2013). During such times, managers should preserve corporate resources within organizations, as investors may perceive discretionary expenditures on philanthropy as a threat to the survival of firms (Muller and Kräussl 2011b). From the perspective of non-trivial costs, corporate philanthropic strategies should become relatively conservative when COVID-19 looms in the local business environment. In a sense, this cost perspective reflects the proverb “prosperity makes friends, adversity tries them.”

The tension between the two perspectives (strategic versus cost) is whether or not firms, as a group, are expected to make social efforts to prevent the deterioration of their local business environment*.* Based on the two perspectives, we develop two competing hypotheses and investigate in the main test how firms, as a group, respond philanthropically to the local spread of the pandemic in their surrounding environment (or specifically, in their headquartered provinces). Methodologically, we construct a balanced panel sample of firm-date data in China and examine whether an increase in the recent provincial number of confirmed COVID-19 cases positively or negatively influences local firms’ decisions to make COVID-19-related donations. As this paper further discusses, the construction of the daily panel sample used in our study, along with the application of the fixed effects model, can largely mitigate the endogeneity issues encountered by prior studies.

It should be noted that among the different levels of analysis classified by Liket and Simaens (2015), our main test falls into the institutional level category, as it focuses on firms’ collective response to the pandemic at the provincial level.^3^ Moreover, unlike prior studies assuming that the features of local environments remain stable over time (e.g., Freeman and Audia 2006; Marquis et al. 2007; Marquis and Battilana 2009; Muller and Whiteman 2009), our paper investigates the impact of COVID-19-induced changes of uncertainty in the local business environment on corporate decisions.^4^ Afterwards, we follow the work of Muller and Kräussl (2011a, b) and investigate investors’ short-term reactions to COVID-19-related donations.

Yet obviously, not all firms have the same philanthropic reactions to the pandemic. Thus, we further analyze whether the variation in the likelihood and amount of COVID-19-related donations can be explained by factors at the organizational level, including pre-existing financial position and reputational and political motives.^5^ Afterwards, we explore the altruistic motive of corporate philanthropy by taking into account the destinations of donations.

The remainder of this paper is organized as follows. We discuss the research background and related literature in Sects. 2 and 3, respectively. Section 4 presents the two competing hypotheses. Section 5 describes the data collection and research design. Section 6 presents the empirical results at the provincial level. In Sect. 7, we further examine the factors affecting philanthropic decisions at the organizational level and explore the implications of donation destinations. Section 8 presents a series of robustness tests. The final section concludes the study and discusses its implications, contributions, and limitations, as well as outlines avenues for future research.

## Background

One of the main reasons for the special investigation into the COVID-19 crisis is the importance of the pandemic. Within months, COVID-19 has spread around the world and become one of the largest pandemics in history (LePan 2020a). As of November 30, 2020, there were 62,363,527 confirmed cases worldwide, including 1,456,687 deaths.^6^ The whole planetary society has been reshaped in lasting ways (POLITICO Magazine 2020). In terms of economic consequences, this pandemic is expected to cause a 5.2% contraction in 2020 global GDP (World Bank 2020). The resulting economic uncertainty is enormous, greater than that associated with the 2008 financial crisis, and resembles the Great Depression of 1929–1933 (Baker et al. 2020).

More importantly, compared with unexpected adverse events such as hurricanes, tsunami, earthquakes, and terrorist attacks, which are isolated events in space and time (Boin and Lagadec 2000), pandemics are contagious and are therefore expected to have a more adverse influence on both donees and donors. For example, the lockdown and restriction policy induced by COVID-19 have prevented most employees from returning to work, thereby disrupting corporate operations. Trading activities involving face-to-face contact have also declined sharply for the same reason. In addition, pandemics tend to last much longer than other natural disasters. What makes COVID-19 worse than previous viruses, such as the 2009 H1N1 and the 2003 SARS, is the lack of vaccine and the speed of contagion (iHeartRadio 2020). The novelty of COVID-19 has immediately led to a global shortage of medical supplies. From a supply-side perspective, corporate philanthropy is expected to decline because operational disruptions should force firms to become tightfisted. From a demand-side perspective, there is strong advocacy for public assistance, especially philanthropic efforts, to solve this crisis. For example, hospitals in Wuhan have asked the public to donate medical supplies, including masks, surgical gowns, and protective clothing.^7^

At first glance, the economic recession triggered by COVID-19 resembles the financial crisis of 2008, the only distinction being that the cause of the COVID-19 crisis is relatively natural, while the 2008 financial crisis was man-made. If so, corporate philanthropic practices should also be similar in the two crises. However, a detailed analysis based on the typology of crises proposed by Gundel (2005) shows fundamental differences between these two crises. According to Gundel (2005), each crisis has two key attributes: (1) predictability, that is, the extent to which the place, time, manner, and probability of the occurrence of a crisis are knowable, and (2) influence possibilities, that is, the extent to which responses to contain or alleviate the crisis are known and possible to execute, before or while it occurs.^8^ The COVID-19 crisis should be both more predictable and influenceable than the 2008 financial crisis, as the public knows the modes of transmission, the risk of becoming infected, and the approaches necessary to prevent the spread of COVID-19. Therefore, firms threatened by the pandemic can take action (e.g., donating medical supplies) to control the spread of the virus, which can potentially accelerate the reopening of the economy. Stated differently, corporate philanthropy is more useful and efficient in dealing with the COVID-19 crisis than the 2008 financial crisis. Overall, the unique features of the COVID-19 crisis, including its nature, extent, and duration, make it important to take a closer look at philanthropic activities during this time.

**Fig. 1:**
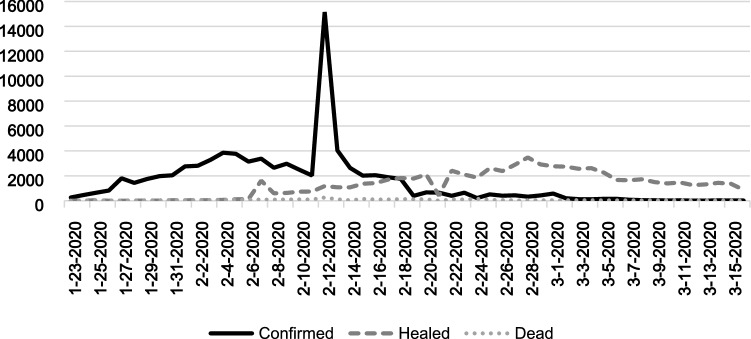
Daily Cases of the Initial COVID-19 Outbreak in China

In several important ways, China provides an opportune and timely setting to analyze the corporate philanthropic response to the pandemic, especially at the early stage. First and foremost, the period for the initial outbreak in China is short, clean, and complete at the time of our study. According to the COVID-19 curves in Fig. 1, the initial outbreak in China exploded in late January 2020 and ended in mid-March. The absence of concurrent mega-events allows us to study corporate actions during an entire outbreak. In contrast, the outbreak curves for many countries and regions, such as the US and the European Union, last much longer than in China (see Appendix B for more details). In particular, the anti-racism protest sparked by the death of George Floyd creates noise in investigations of the pandemic’s impact on corporate decisions in the US. Second, the need for public participation in tackling the crisis is relatively high in China. For example, the shortage of face masks in China should last longer than in countries without a country-wide face mask wearing policy (Wu et al. 2020a, b). Compared with other countries, which have had time (for most countries, the time lag is more than one month) to get prepared and learn from China’s experience (e.g., Cyranoski 2020), China was the first country to experience the COVID-19 outbreak, so its preparedness for this virus should be relatively low. In addition, the consistently low level of health security in China, based on the 2019 Global Health Security Index (LePan 2020b), makes it difficult to rely solely on the government to solve the shortage of medical supplies. These features of the Chinese setting have led to many calls for help, as described in Footnote 8. Third, the COVID-19-related policies in China were unified and applied nationwide, making the economic consequences of the pandemic comparable across provinces.^9^ Finally, as the government controls key resources in China, Chinese firms have strong political incentives to proactively engage in philanthropic activities (e.g., ARCP 2007; Wang and Qian 2011). For this reason, similar to the reactions to the 2008 Wenchuan earthquake, Chinese firms are expected to support the government by immediately donating the resources in shortage. Therefore, this country-specific feather of the Chinese setting intensifies the tension between the competing perspectives and thus increase the necessity of our study.

## Literature Review

### Determinants of Corporate Philanthropy

Although it takes various forms, corporate philanthropy usually refers to the private giving of time or resources for public purposes (Salamon 1992). According to the widely accepted CSR pyramid model proposed by Carroll (1979; 1991), corporate philanthropy is the most discretionary subset of CSR activities. Scholars have also identified a number of factors determining corporate philanthropic decisions. According to Liket and Simaens (2015), these determinants can be systematically classified into three levels: individual, organizational, and institutional. In addition to the institutional level defined in Footnote 4, determinants at the individual level capture the “decision-making processes, preferences, or actions of individuals” (Liket and Simaens 2015, p. 288), while those related to “organization as an entity” (Liket and Simaens 2015, p. 288) belong to the organizational level. Similarly, Gautier and Pache (2015) classify determinants of corporate philanthropy into individual, firm level, and field level.

One of the important institutional constituents affecting corporate philanthropy is the surrounding environment of a firm (e.g., Useem 1988; Marquis et al. 2007; Muller and Whiteman 2009; Tilcsik and Marquis 2013; Gautier and Pache 2015; Liket and Simaens 2015). The scope of the environment investigated in the literature ranges from the national level to the community level. Many studies analyze the interactions between firms and their stakeholders in the local environment. Specifically, because the local environment, within which firms must operate, has a significant and long-standing influence on firm decisions, firms need to form ongoing relationships with related stakeholders for their survival and future growth (e.g., Godfrey 2005; Marquis and Battilana 2009). In this regard, firms are motivated to take social actions that will help them accrue social capital in the local environment (Godfrey 2005; Husted and de Jesus Salazar 2006). This perspective is known in the corporate philanthropy literature as strategic philanthropy (e.g., Porter and Kramer 2002; Saiia et al. 2003; Maas and Liket 2011; Gautier and Pache 2015; Liket and Simaens 2015; Cyr 2018). *According to *Porter and Kramer (2002)*, corporate social actions, if strategically implemented, can be integrally connected to the economic objectives of firms.* The strategic perspective is extensively used to explain “home region bias” in philanthropy. For example, in the analysis of Fortune Global 500 firms’ donation announcements to the South Asian tsunami, Hurricane Katrina, and the 2005 Kashmir earthquake, Muller and Whiteman (2009) find evidence that a firm is more likely to donate in terms of frequency and amount if it is located in the disaster-stricken region. This geographic pattern is attributed by the authors to firms’ “long standing links in the region” (Muller and Whiteman 2009, p. 593), also referred to as “local particularities” in other studies (e.g., Marquis and Battilana 2009, p. 283). As our study focuses on the spread of the pandemic in the local business environment, the most relevant studies are those related specifically to adverse events in the local environment. We separately discuss these studies in the following subsection.

A key stakeholder in the local environment is the government, which can significantly influence corporate philanthropic practices through business intervention (or alternatively, institutional pressure). For example, Bertrand et al. (2020) find that Fortune 500 and S&P 500 firms seek tax exemption through philanthropic activities. This type of pressure is particularly prominent in emerging markets (e.g., Marquis and Raynard 2015), leading a series of studies to investigate how corporate philanthropy is further shaped by this unique feature. In this regard, many studies find similar evidence that firms aim to build connections with authorities and seek political favors through corporate philanthropy (e.g., Sánchez 2000; Su and He 2010; Wang and Qian 2011; Jia and Wang 2013; Li et al. 2015; Kim 2017; Bertrand et al. 2020; Hao et al. 2020; Yang and Tang 2020). For example, Wang and Qian (2011) find evidence that corporate philanthropy enhanced the corporate financial performance of Chinese firms from 2001 to 2006 and conclude that philanthropic activities help firms to elicit positive stakeholder responses and gain political access. Furthermore, even within the same emerging market, different firms (e.g., politically connected versus non-connected) have different levels of institutional pressure and political incentives, leading to further variation in philanthropic decisions.

Reputational incentive is another important determinant of philanthropic activities. A widely accepted perspective is that corporate philanthropy builds reputation (e.g., Fombrun and Shanley 1990; Smith 1994; Brammer and Millington 2005; Godfrey 2005), which can potentially increase customers’ product purchase intentions (Strahilevitz and Myers 1998; Sen and Bhattacharya 2001). As such, many studies support that corporate philanthropy can be used as a strategic form of advertisement to gain competitive advantage and promote sales (Navarro 1988; Brown et al. 2006; Lev et al. 2010; Gao et al. 2012; Zhang et al. 2010a, b, c). This reputational motive can be further reinforced by public and peer pressure, especially in the case of catastrophic events (Zhang et al. 2010a, b, c). In addition, as the moral capital accumulated through corporate philanthropy can act as insurance-like protection against business risks (Godfrey 2005), philanthropy can be used as a reputation crisis management tool (i.e., to cover “wrongdoing”). For example, in a sample of 384 US companies, Koehn and Ueng (2009) find that firms with restated earnings are more generous in their corporate giving. Overall, consistent with the strategic perspective of corporate philanthropy, all of the aforementioned determinants suggest that corporate social giving leads to having.

However, as philanthropy is essentially a costly expense and represents a direct outflow of resources (Fombrun et al. 2000), a firm’s philanthropic decisions also depend on its resource availability. This perspective is known as slack resources theory in the literature (e.g., Waddock and Graves 1997; Buchholtz et al. 1999; Orlitzky and Benjamin 2001; Dunn 2004; Seifert et al. 2004). There are various measures for slack resources. For example, Waddock and Graves (1997) use prior financial performance as an indicator of resource availability and find that it is positively associated with corporate social performance. Seifert et al. (2004) consider cash flow as one of the most discretionary types of organizational slack and find that this type of slack significantly influences corporate cash donations. Crampton and Patten (2008) find that changes in corporate contributions after 9/11 are positively and significantly associated with pre-existing short-term profitability (ROA). Although slack resources theory suggests that having leads to giving, it does not necessarily contradict the aforementioned strategic view. Rather, the relationship between corporate social performance and financial performance can be painted as a virtuous cycle, in which social performance simultaneously depends on prior financial performance and affects subsequent financial performance.

Etymologically, there is a benevolent nature in philanthropy, as it means “love of humanity” in Greek. Thus, many studies also consider the altruistic motive as an important driver of philanthropy in addition to the various for-profit motives (e.g., Fry et al. 1982; Gan 2006; Eger et al. 2019). A survey study conducted by Sargeant and Stephenson (1997) shows that most businesses do not seek any gain from their charitable support. Campbell et al. (1999) confirm that corporate giving is motivated by a sense of social responsibility. The altruistic motive may come either from a firm’s managers or from the firm as an entity. At the individual level, corporate philanthropy may be the result of managers’ personal values (Hemingway and Maclagan 2004), such as benevolence and integrity (Choi and Wang 2007), and their perceived moral obligation (Dennis et al. 2009). At the organizational level, Muller et al. (2014) develop a framework in which managers’ philanthropic decisions reflect the collective empathy of members of the organization.

### Corporate Social and Philanthropic Responses to Adversity

It is of great value to separately review studies of corporate philanthropic response to natural disasters, such as earthquakes (e.g., Zhang et al. 2010a, b, c; Zhang et al. 2010a, b, c; Gao 2011; Gao et al. 2012; Li et al. 2015), tsunami (e.g., Patten 2008; Muller and Whiteman 2016), hurricanes (e.g., Muller and Kräussl 2011a, b), and terrorist attacks (e.g., Crampton and Patten 2008). Many of these studies are consistent with the strategic perspective of corporate philanthropy. For example, Patten (2008) shows that donation announcements by 79 US firms to the 2004 tsunami in Southeast Asia triggered positive short-term market reactions. Similarly, Gao et al. (2012) find that donation announcements to the 2008 Wenchuan earthquake were associated with positive market reactions in China, but mainly for firms providing products (or services) directly to consumers.

However, opposing views claim that the influence of disasters on corporate operations should be taken into account. According to Godfrey et al. (2009), some disasters may impose non-trivial costs for firms. Muller and Kräussl (2011a) focus specifically on Hurricane Katrina and argue that this event created significant economic uncertainty, which prompted investors to reassess firm value. Their empirical results confirm that investors indeed reacted negatively to the shock of Hurricane Katrina, especially for firms seen as socially irresponsible. Moreover, they provide evidence that socially irresponsible firms were more likely to make Katrina-related donations, potentially because of the desire to improve their reputation. In addition to the negative reactions to Hurricane Katrina, Muller and Kräussl (2011b) find that investors reacted negatively to Katrina-related donation announcements. Their main explanation is that the uncertainty surrounding Hurricane Katrina led investors to believe that philanthropy at this time could threaten firms’ economic continuity. The most important insight that we can draw from these two studies is that disaster-induced economic uncertainty can have real effects, for example, on investors’ assessment of firm value and firms’ philanthropic decisions during these events. The extent of the real effects depends on the severity of the damage caused by the adverse events. In this area, Tilcsik and Marquis (2013) adopt an institutional perspective similar to ours and find that in the year when natural disasters strike the local communities of Fortune 1000 firms, there is no sign of fluctuation in philanthropic donations, but donations from these firms increase in the year following small disasters and decrease in the year following major disasters.^10^ The non-trivial costs of adverse events can also influence other corporate social decisions. For instance, Pondeville et al. (2013) find that in times of environmental uncertainty, firms are less likely to adopt a proactive environmental strategy, suggesting that firms tend to step back and adopt a defensive strategy in difficult times.

Yet it remains debatable whether the hurricane-type of disasters will impose nationwide uncertainty. Dessaint and Matray (2017) find that firms located in the neighborhood of the hurricane-stricken area, even though they are not directly affected by the disaster, increase their cash holdings to express concerns about hurricane risk in 10-Ks/10-Qs. Specifically, they posit that if the induced economic uncertainty spills over from affected areas to neighboring areas, there should be a reduction in investment or an increase in the variance of revenue in neighboring firms. However, they find no evidence to support this point, thereby interpreting their results as managers’ overreactions and ruling out the explanation of local economic uncertainty. One common implication of this study and that of Muller and Kräussl (2011b) is that corporate decisions in response to adverse events are seemingly irrational.

We also seek insight from related studies on the 2008 financial crisis. Back then, many media voices urged firms to cut back on philanthropic giving and spend only on core business activities (e.g., Franklin 2008; Munoz 2009). There is statistical support for the decline in philanthropy during this period. For instance, from 2007 to 2008, the total amount of charitable donations dropped by around 7% in the US (Reich and Wimer 2012). But other than that, studies in this area focus primarily on overall CSR activities and discuss whether the 2008 financial crisis will end CSR activities, rather than paying special attention to corporate philanthropy. For example, Manubens (2009) claims that the decrease in product demand and consumers’ fixation on low prices during the financial crisis could prompt firms to race to the bottom, that is, to bypass social compliance and gain a comparative cost advantage. According to Karaibrahimoglu (2010), as the financial crisis forced firms to lay off employees, postpone investments, and cut subsequent budgets, CSR spending was unlikely to take priority over key operating activities. Using paired samples t-tests with 100 randomly selected Fortune 500 firms, he finds a significant drop in the number and scale of CSR projects during the financial crisis. Likewise, Kemper and Martin (2010) show that during this difficult time, firms were more likely to conserve corporate resources and save them as “seed corn” for future operations. Overall, these studies are consistent with the aforementioned perspective of non-trivial costs.

On the opposite side, some studies from the strategic perspective *share the idea that during a crisis, rather than threatening firm survival, CSR can actually become an opportunity for firms to relocate their business to a better position (*Souto 2009; Yelkikalan and Köse 2012*). From this perspective, the value created by CSR activities may not only mitigate the negative short-term economic effects of financial crises, but also help to differentiate products or services and strengthen relationships with stakeholders, especially customers (e.g., *Giannarakis and Theotokas 2011*), which in turn sustain long-term profit (e.g., *Karaibrahimoglu 2010*). On this side, there are some studies providing supporting evidence. For instance, *Giannarakis and Theotokas (2011)* use the Global Report Initiative (GRI) guidelines to evaluate the CSR reports of 112 firms between 2007 and 2010 and find better CSR performance for these firms before and during the 2008 financial crisis than for the 2009–2010 period. Similarly, *García-Benau et al. (2013)* find a significant increase in the number of CSR reports in Spain after the 2008 financial crisis. In terms of CSR outcomes, *Selvi et al. (2010)* document a positive association between CSR and firm reputation in Turkey before and after the 2008 financial crisis. *Arevalo and Aravind (2010)* focus on firms’ compliance with a key CSR initiative (the United Nations Global Compact [GC]) and find that the most compliant firms were less affected by the financial crisis. The survey study conducted by *Harwood et al. (2011)* shows that most respondents, who held senior positions (chief executives, managing directors, directors, or senior managers) in UK-based organizations, were satisfied with their organizations’ current CSR efforts and forecasted an increase in both environmentally and socially responsible activities. They interpret these results as confirmation of the resilience of CSR activities during economic recessions. However, it should be noted that the shortcoming of these studies is their lack of rigid empirical support.*

## Hypothesis Development

Based on the above literature review, it remains controversial whether corporate philanthropy is resilient in times of crisis. While the economic concern induced by COVID-19 may force firms to tighten or even cease their discretionary expenditures on philanthropy, in reality, many firms have proactively made official philanthropic commitments and taken steps to support the fight against COVID-19. Essentially, this controversy boils down to the question of whether firms’ social objectives can be aligned with their economic objectives (Maas and Liket 2011). To examine the exact effect of the COVID-19 crisis on corporate philanthropy, we propose two competing hypotheses based on the perspectives of strategic and non-trivial costs.

### Philanthropic Response from the Strategic Perspective

The strategic nature of corporate philanthropy, often referred to as “strategic philanthropy,” is widely accepted in the literature (e.g., Porter and Kramer 2002; Saiia et al. 2003; Maas and Liket 2011; Gautier and Pache 2015; Liket and Simaens 2015; Cyr 2018). From this perspective, firms’ social and economic objectives are not necessarily in conflict and can even be integrally connected (Post and Waddock 1995; Saiia et al. 2003; Porter and Kramer 2002; Cyr 2018). When carried out strategically, corporate philanthropic activities have “the potential to result in a win–win situation with positive impact on both social welfare and profitability” (Maas and Liket 2011, p. 445). One example used by Porter and Kramer (2002) is that improving the education level of the local workforce, which is a social issue, can potentially increase a company’s competitiveness. Likewise, while the fight against COVID-19 is a social issue, short-term philanthropic donations made by firms to protect the local business environment can potentially be economically beneficial in the long term.

This type of corporate philanthropy can be seen as an investment to improve the business environment (Gautier and Pache 2015). Firms cannot thrive in a degraded environment because their ability to compete strongly depends on the quality of the surrounding business environment (Porter and Kramer 2002; Gautier and Pache 2015). As such, philanthropic efforts to prevent the deterioration of the local business environment can positively influence firm competitiveness in the long run. Moreover, during difficult times, the propensity of firms to invest in the local business environment should be relatively high due to the lack of other investment opportunities (Dessaint and Matray 2017). It is therefore not surprising that in the face of increased environmental uncertainty, some firms might adopt a proactive strategy to reduce the uncertainty (Pondeville et al. 2013).

In China, an increase in COVID-19 cases in each province will immediately trigger official reactions, such as the activation of Level-1 public health emergency and implementation of restriction and lockdown, which significantly affects corporate operations.^11^ As discussed earlier, compared with other adverse events, such as natural disasters and the 2008 financial crisis, the COVID-19 crisis is more predictable and influenceable, as the public knows the modes of transmission of the virus, the risk of becoming infected, and the approaches necessary to control the spread of the virus. Therefore, in their own interests, firms may take proactive strategies to limit the speed and extent of local pandemic spread, rather than reactively complying with pandemic-related policies and regulations. To prevent the COVID-19-induced deterioration of local business environment, the easiest and most effective action firms can take is to resolve the shortage of cash and medical supplies through donations, as most other volunteer activities are severely constrained by the restriction and lockdown policy. Moreover, as the contagious virus can easily spread out from affected regions (mainly Hubei Province), donations to these regions may be as crucial as donations to the local business environment. Overall, from the strategic perspective, if firms jointly donate the necessary resources to fight COVID-19, they are expected to suffer less from this pandemic.

Besides, firms can use philanthropy to accrue intangible benefits, which can potentially improve their competitive advantage and provide long-term benefits (e.g., Fombrun and Shanley 1990; Brammer and Millington 2005; Godfrey 2005). A series of studies show that corporate philanthropy can be used as a tool to build connections with authorities and seek political favors (Sánchez 2000; Su and He 2010; Wang and Qian 2011; Jia and Wang 2013; Li et al. 2015; Kim 2017; Hao et al. 2020; Yang and Tang 2020). This political incentive should be particularly strong in China, where the government still holds enormous regulatory power over resource allocation. Also, corporate philanthropy can be strategically used as a special form of advertisement to promote sales (Navarro 1988; Brown et al. 2006; Lev et al. 2010; Gao et al. 2012; Zhang et al. 2010a, b, c). Critically, even within the same environment, not all firms have the same level of incentive to engage in strategic philanthropic activities (e.g., Wang and Qian 2011). Taking these findings together, we expect that firms’ decisions to make COVID-19-related donations should also reflect the firm-level variations in political and reputational motives.

Although altruism may not be a good explanation for philanthropy based on self-interest (Harbaugh 1998), especially during difficult times, we do not exclude the coexistence of an altruistic explanation. The same view is adopted by Muller and Kräussl (2011a). Adverse events (e.g., earthquakes, hurricanes, tsunami) can generate empathy among members of an organization (Weiss and Cropanzano 1996), leading to a desire to improve the well-being of others outside the organization (Batson et al. 2007; Muller et al. 2014). Likewise, during the COVID-19 crisis, media coverage of victims’ stories and the sacrifices of medical staff and volunteers has generated empathy across society. For example, there have been many emotional media reports, such as the death of an entire family (Graham-Harrison 2020) and infected doctors who voluntarily joined the fight against the pandemic (Su 2020). Accordingly, the altruistic perspective also implies that corporate philanthropy should increase with the spread of the pandemic.

In short, from the strategic perspective, corporate philanthropy can help both society as a whole and donating firms find a pathway out of the crisis. This alignment of firms’ social and economic objectives should lead to a collective corporate philanthropic response to the spread of COVID-19 in the local business environment. At the organizational level, corporate philanthropic decisions vary according to their political and reputational motives. Even taking altruism into account, corporate philanthropy is still expected to increase with the severity of the local pandemic spread. Therefore, we propose the following hypothesis:

## H1a: Corporate philanthropic response to the COVID-19 crisis increases with the severity of the local spread of the pandemic.

### Philanthropic Response from the Cost Perspective

However, scholars also suggest the existence of constraints on the alignment of economic and social goals (e.g., Husted and de Jesus Salazar 2006). Two types of costs should be taken into account in corporate philanthropic decisions during adverse events. The first type is corporate philanthropy per se, which has long been seen as competing with firms’ economic objectives (Hillman and Keim 2001; Porter and Kramer 2002). Whatever the motives, the prerequisite for corporate philanthropy is that a firm must have sufficient resources. This is commonly referred to as slack resources theory in the literature (e.g., Waddock and Graves 1997). Following this reasoning, prior studies find supporting evidence that firm-level variations in corporate resources (e.g., ROA, cash holdings) significantly affect corporate philanthropic decisions (e.g., Waddock and Graves 1997; Adams and Hardwick 1998; Seifert et al. 2004; Crampton and Patten 2008). The second type is the non-trivial costs imposed by adverse events on all firms in the affected region (Godfrey et al. 2009). For example, the non-trivial costs of Hurricane Katrina include the costs of “supply chain disruptions, employee strain, deteriorated employee performance, the diversion of managerial and employee attention” (Muller and Kräussl 2011b, p. 204). Because all firms in the hurricane-stricken area are affected, this type of cost should translate into economic uncertainty in the affected area (e.g., Muller and Kräussl 2011a, b; Dessaint and Matray 2017).

Compared with other disasters (e.g., hurricanes, tsunami, earthquakes, and terrorist attacks) isolated in space and time (Boin and Lagadec 2000), COVID-19 has undoubtedly caused more economic uncertainty in terms of both duration and extent. Altig et al. (2020) examine a series of indicators of economic uncertainty and find that all indicators show huge jumps in response to COVID-19.^12^ In China, increased economic uncertainty is particularly reflected in changes in COVID-19-related policies. For example, as COVID-19 cases declined in April, the Beijing government allowed students to return to school (Khaliq 2020), while the resurgence of the pandemic in June forced the Beijing government to raise its COVID-19 alert level and extend the lockdown policy (DW News 2020).

To adapt to increased economic uncertainty, firms can switch to cost containment strategies. During such times, investors are reluctant to accept philanthropic decisions, as they expect that “managers’ primary responsibility at such times is one of economic continuance” (Muller and Kräussl 2011b, p. 205). Corporate resources should be preserved in the organization, either as a buffer against potential liquidation risk (Dessaint and Matray 2017) or as “seed corn” for future operations (Kemper and Martin 2010). Overall, in the interests of shareholders, firms should cut the budget for philanthropy to support their survival in times of adversity.

Taken together, from the perspective of non-trivial costs, the local spread of COVID-19 is expected to increase local economic uncertainty, forcing firms in the local business environment to become conservative in their resource allocation decisions and save the cost of philanthropic spending. This leads to the opposite hypothesis:

## H1b: Corporate philanthropic response to COVID-19 decreases with the severity of the local spread of the pandemic.

## Research Design

### Sample Selection and Data

Our initial sample consists of all Chinese firms listed on the Shanghai and Shenzhen stock exchanges at the end of 2018.^13^ Corporate financial data are obtained from the China Stock Market & Accounting Research (CSMAR) Database. We choose a 7-week sample period, starting on January 23, 2020, when information on the number of COVID-19 cases in each province and city became public and the lockdown policy was implemented in Wuhan city (and nearby cities),^14^ and ending on March 11, when the pandemic was mainly under control in China (see the COVID-19 curve in Fig. 1). To measure each firm’s donations in relation to the daily COVID-19 data during these 49 days, we construct a balanced panel sample of firm–date data. This approach yields a final sample of 176,645 observations (3,605 firms over 49 days).

There is little difference between the COVID-19 data offered by different Chinese media platforms, as they are all based on data provided by the National Health Commission of the PRC. As we focus on the response to the observed number of COVID-19 cases, data provided by social media are more suitable than official data because social media platforms are more exposed to the public. Therefore, we derive our COVID-19 data from Tencent’s search engine, a leading Internet company in China.^15^ We use an R package named *nCov2019* developed by Wu et al. (2020a; b) to obtain daily COVID-19 data in each province, including the number of confirmed, recovered, and deceased cases. These provincial COVID-19 data are then matched with each firm based on the location of its headquarter.^16^

To identify COVID-19-related donations of cash and supplies, we follow the procedures below. First, we manually collect data on COVID-19-related donations from all available sources, including corporate disclosures and media news.^17^ Second, for each donation, we manually search for the earliest news report date (or announcement date for corporate disclosures)^18^ and the date of actual actions. The donation date is defined as the earlier of the two dates.^19^ Afterwards, for each firm in each day, we separately identify the amount of cash donations and the estimated amount of donated supplies.^20^ For example 1 in Appendix A, we can get the following information based on the above procedures: RMB 5 million in cash and RMB3.35 million in supplies on February 4, 2020, RMB5 million in cash on January 26, and a total of RMB 9 million in cash and supplies on January 31.

### Regression Model and Main Variables

We use the following fixed effects model to test the two competing hypotheses:

1 $${Donation}_{it}=\alpha {Confirm}_{[-3,-1]}+Firm\_FE+Date\_FE,$$

where *Donation*_*it*_ is the donation choice made by firm *i* at date *t*, measured by *Don_Dum* and *Don_Amt*. *Don_Dum* is a dummy variable indicating whether a firm donates.^21^*Don_Amt* stands for the donation amount, calculated as the natural logarithm of one plus the amount of cash donated and the estimated value of donated supplies. *Confirmed*_*[−3, −1]*_ is the main independent variable, which measures the severity of the local spread of the pandemic and is calculated as the natural logarithm of one plus the total number of confirmed cases between *t* *−* 1 and *t* *−* 3 in the province where each observed firm is headquartered. The coefficient of *Confirmed*_*[−3,−1]*_ is expected to be positive under the strategic hypothesis and negative under the cost hypothesis.

Note that Model (1) also includes firm and date fixed effects (*Firm_FE* and *Date_FE*, respectively). In essence, this is a DID (difference-in-differences) approach. *Date_FE* controls for daily characteristics. For example, there may be a cluster of donations around a certain date due to political encouragement or peer pressure. *Firm_FE* controls for firm, industry, and regional characteristics.^22^ Besides, most common control variables used in the literature, such as firm size or leverage, remain unchanged during our sample period and are thus omitted. Overall, endogeneity is largely mitigated in this method.

### Descriptive Statistics

Among the 3605 firms listed on the A-share market, 885 made donations on different dates during the sample period. Panel A of Table 1 presents the descriptive statistics. The mean of *Don_Dum* is 0.005, suggesting that the average probability for a firm to donate in one day is 0.005. For both *Don_Amt* and *Confirmed*_*[−3, −1]*_, we use the log-transformed variables in the regression. The statistics for *Don_Amt* indicate that the highest donation amount made in one day is RMB62 million [(e^8.732–1^ *−* 1) × 10000]. Due to the very low donation percentage, the mean amount is only RMB335 [(e^0.033^ – 1) × 10000]. According to the statistics for *Confirmed*_*[-3,-1]*_, the 3-day provincial number of confirmed cases ranges from 0 to 3849 [e^8.256^ – 1]. Panel B shows the geographic distribution of the full sample. Basically, firms headquartered in developed provinces, such as Guangdong and Beijing, were more likely to make donations than those in Western China.

**Table 1:** Descriptive Statistics

*Variable*	*N*	*Mean*	*Std. Dev*	Min	P25	Median	P75	Max
Panel A: Descriptive Statistics								
* Don_Dum*	176,645	0.005	0.072	0.000	0.000	0.000	0.000	1.000
* Don_Amt*	176,645	0.033	0.456	0.000	0.000	0.000	0.000	8.732
* Confirmed*_*[*_***−***_*3,*_***−***_*1]*_	176,645	2.675	1.954	0.000	0.693	2.708	4.248	8.256

Province	No. of observations in the full sample	Percentage (%) of observations in the full sample	No. of donation observations	Percentage (%) of donation observations
Panel B: Geographic Distribution of the Sample				
Anhui	4,949	2.802%	30	3.240%
Beijing	19,061	10.791%	102	11.015%
Chongqing	2,303	1.304%	19	2.052%
Fujian	6,468	3.662%	43	4.644%
Gansu	1,470	0.832%	15	1.620%
Guangdong	29,351	16.616%	147	15.875%
Guangxi	1,617	0.915%	9	0.972%
Guizhou	1,372	0.777%	17	1.836%
Hebei	2,646	1.498%	22	2.376%
Heilongjiang	1,421	0.804%	10	1.080%
Henan	3,871	2.191%	23	2.484%
Henan	1,323	0.749%	14	1.512%
Hubei	4,606	2.607%	36	3.888%
Hunan	4,949	2.802%	28	3.024%
Inner Mongolia	1,078	0.610%	9	0.972%
Jiangsu	19,502	11.040%	47	5.076%
Jiangxi	2,009	1.137%	17	1.836%
Jilin	1,666	0.943%	16	1.728%
Liaoning	3,381	1.914%	20	2.160%
Ningxia	588	0.333%	4	0.432%
Qinghai	441	0.250%	2	0.216%
Shaanxi	2,401	1.359%	11	1.188%
Shandong	9,457	5.354%	61	6.587%
Shanghai	15,729	8.904%	58	6.264%
Shanxi	1,617	0.915%	11	1.188%
Sichuan	5,831	3.301%	38	4.104%
Tianjin	2,058	1.165%	10	1.080%
Tibet	686	0.388%	2	0.216%
Xinjiang	2,597	1.470%	15	1.620%
Yunnan	1,617	0.915%	9	0.972%
Zhejiang	20,580	11.650%	81	8.747%

## Main Results

### Corporate Philanthropic Response to the Local Spread of COVID-19

Table 2 presents the regression results for the corporate philanthropic response to the local spread of the pandemic. The dependent variable is *Don_Dum* in Column (1) and *Don_Amt* in Column (2). In Column (1), the coefficient of *Confirmed*_*[-3,-1]*_ is significant and negative (-0.001, with a *t*-value of -2.58). In Column (2), we replace *Don_Dum* with *Don_Amt* and still find a negative coefficient of *Confirmed*_*[-3,-1]*_ (-0.004, with a *t*-value of -2.87). The results show that when the number of confirmed COVID-19 cases in the previous three days increases in a province, firms headquartered in this province are less likely to donate in terms of both likelihood and amount. In other words, at the provincial level, the deterioration of pandemic spread makes firms tightfisted in donations. This negative philanthropic response at the provincial level supports the cost perspective of philanthropy.

**Table 2:** Corporate Philanthropic Response to the Local Spread of COVID-19

	(1)		(2)
*Dep. Var.* =	*Don_Dum*		*Don_Amt*
***Confirmed***_***[−3,−1]***_	− 0.001***		− 0.004***
	**(− 2.58)**		**(− 2.87)**
Constant	0.007***		0.045***
	(10.76)		(10.88)
Firm fixed effects	Yes		Yes
Date fixed effects	Yes		Yes
Obs	176,645		176,645
Adj. *R*^*2*^	0.010		0.011

The *t*-values (in parentheses) are based on heteroskedasticity-consistent standard errors clustered by firm

***, **, and * indicate significance at the 1%, 5%, and 10% levels, respectively (two-tailed)

Variables of interest are marked in bold

### Investor Reactions to COVID-19-Related Philanthropic Donations

An implication of the above results is that during difficult times, firms generally perceive philanthropy as a threat to their survival. Following Muller and Kräussl (2011a, b), we also explore whether investors hold similar perceptions and react negatively to philanthropy during the pandemic.

We use the cumulative abnormal returns (CARs) from date *t* to *t* + *2* as the dependent variable (*CAR*_*[0,*+*1]*_). Daily donation decisions (i.e., *Don_Dum* and *Don_Amt*) and their interaction with *Confirmed*_*[-3,-1]*_ are included as independent variables. Table 3 shows the results. The smaller sample is due to the suspension of stock trading during the Chinese Spring Festival.

**Table 3:** Investor Reactions to Corporate Philanthropy During COVID-19

	(1)	(2)	(3)	(4)
*Dep. Var.* =	*CAR[0,* + *2]*	*CAR[0,* + *2]*	*CAR[0,* + *2]*	*CAR[0,* + *2]*
***Don_Dum***	− 0.010***	**0.000**		
	**(− 2.96)**	**(0.04)**		
***Don_Dum × Confirm***_***[−3,−1]***_		− 0.004**		
		**(− 2.19)**		
***Don_Amt***			− 0.002***	** − 0.001**
			**(− 2.86)**	**(− 0.67)**
***Don_Amt × Confirm***_***[−3,−1]***_				− 0.000*
				**(− 1.71)**
***Confirmed***_***[−3,−1]***_	− 0.001***	− 0.001***	− 0.001***	− 0.001***
	**(− 3.46)**	**(− 3.47)**	**(− 3.46)**	**(− 5.46)**
Constant	− 0.001	− 0.000	− 0.001	− 0.001*
	(− 1.18)	(− 0.70)	(− 1.18)	(− 1.83)
Firm fixed effects	Yes	Yes	Yes	Yes
Date fixed effects	Yes	Yes	Yes	Yes
Obs	103,293	103,293	103,293	103,293
Adj. *R*^*2*^	0.094	0.080	0.094	0.094

The *t*-values (in parentheses) are based on heteroskedasticity-consistent standard errors clustered by firm

***, **, and * indicate significance at the 1%, 5%, and 10% levels, respectively (two-tailed)

Variables of interest are marked in bold

First, the negative coefficients of *Confirmed*_*[-3,-1]*_ in all of the columns suggest that the number of local COVID-19 cases constantly changes investors’ evaluation of firm value. Second, in Columns (1) and (3), the negative coefficients of *Don_Dum* and *Don_Amt* (-0.010 and -0.002, with *t*-values of -2.96 and -2.86, respectively) suggest that investors react negatively to COVID-19-related donations. In addition, as indicated by the negative coefficients of the interaction term in Columns (2) and (4), investor reactions to COVID-19-related donations become more negative as the local pandemic situation worsens. Our interpretation is that investors expect local firms to experience more negative economic consequences when the local spread of the pandemic worsens, prompting them to question the merits of philanthropy during this period. The above results echo the work of Muller and Kräussl ([Bibr CR71][Bibr CR72]) that the economic consequences imposed by adverse events can “engage investors in active sensemaking and reevaluation of a firm” (Pfarrer et al. 2010, p. 1133). The results in this subsection also highlight investors’ sensitivity to key information about adversity in their reassessment process.

### Further Analyses on Organizational Variation in Philanthropic Decisions

The results in Table 2 indicate that for local firms as a group, concern for economic uncertainty outweighs the strategic incentive of philanthropic decisions when their business environment is hit by the pandemic. However, this negative response at the provincial level, or at the institutional level based on the classification by Liket and Simaens (2015), does not entail that all firms behave in the same way. On the one hand, variation in resource availability determines the extent to which firms perceive the cost of philanthropy as a threat to their operations. On the other hand, different firms have different levels of political and reputational incentives. In cases when the strategic value of philanthropy outweighs the aggregate amount of philanthropic cost and the non-trivial costs imposed by the pandemic, firms should be more likely to lend a helping hand to society. Therefore, in this section, we analyze in detail how factors at the organizational level further affect firms’ response to the pandemic.

### Pre-existing Resource Availability and Corporate Philanthropic Response to COVID-19

Based on the slack resources theory discussed in the cost perspective, we conduct the following analyses to examine whether the corporate philanthropic response is further affected by organizational variation in pre-existing resource availability. Table 4 shows the results.

**Table 4:** Pre-existing Resource Availability and Corporate Philanthropic Response

	(1)	(2)	(3)	(4)
*Dep. Var.* =	*Don_ Dum*	*Don_Amt*	*Don_Dum*	*Don_Amt*
*Panel A*				
*Confirmed*_*[−3,−1]*_	− 0.001***	− 0.005***	− 0.001***	− 0.006***
	(− 2.86)	(− 3.20)	(− 3.22)	(− 3.64)
***Confirmed***_***[−3,−1]***_** × Healthy**	0.002***	0.015***		
	**(3.09)**	**(3.49)**		
***Confirmed***_***[−3,−1]***_** × HighCash**			0.002***	0.018***
			**(4.39)**	**(5.07)**
Constant	0.007***	0.044***	0.007***	0.043***
	(10.63)	(10.73)	(10.28)	(10.32)
*Panel B*				
***Confirmed***_***[−3,−1]+***_ ***Confirmed***_***[−3,−1]***_** × Healthy**	0.001*	0.011**		
*F − Value*	**3.69**	**4.98**		
***Confirmed***_***[−3,−1]+***_ ***Confirmed***_***[−3,−1]***_** × HighCash**			0.001***	0.012***
*F − Value*			**7.17**	**10.28**
Firm fixed effects	Yes	Yes	Yes	Yes
Date fixed effects	Yes	Yes	Yes	Yes
Obs	176,645	176,645	176,645	176,645
Adj. *R*^*2*^	0.010	0.011	0.011	0.011

The *t*-values (in parentheses) are based on heteroskedasticity-consistent standard errors clustered by firm

***, **, and * indicate significance at the 1%, 5%, and 10% levels, respectively (two-tailed)

Variables of interest are marked in bold

In Columns (1) and (2), we use the *Z*_*China*_ score developed by Zhang et al. (2010a, b, c) to measure corporate financial health. Firms with a *Z*_*China*_ score in the top decile at the end of 2019 are defined as financially healthy firms (*Healthy*). In Panel A, we can see that the coefficients of the interaction between *Confirmed*_*[-3,-1]*_ and *Healthy* are significant and positive, suggesting that financially healthy firms are more likely to make donations than distressed firms when the pandemic worsens.

Alternatively, in Columns (3) and (4), we use corporate cash holdings to measure resource availability. Specifically, *HighCash* indicates whether corporate cash holdings at the end of 2019 are in the top decile. Firms with high cash holdings are more likely to have slack resources and are therefore less likely to worry about their survival during adverse events. Compared with firms that experience cash shortages, these firms should have more freedom to allocate resources to tackle social problems. In Panel A, while the coefficients of *Confirmed*_*[-3,-1]*_ remain negative, the positive coefficients of the interaction term between *Confirmed*_*[-3,-1]*_ and *HighCash* suggest that high cash holdings before the pandemic mitigate the negative philanthropic response to the local pandemic spread. In fact, based on the results in Panel B of Table 4, firms with abundant resources are even more likely to make COVID-19-related donations. Taken together, the results in Table 4 show the importance of resource availability on each firm’s philanthropic response to the pandemic.

### Political Motive and Corporate Philanthropic Response to COVID-19

Political considerations may also influence philanthropic decisions. Some firms have strong incentives to gain government support and nurture political connections, which can be converted into future profitability, especially in countries where property rights are underdeveloped (Sánchez 2000; Su and He 2010; Gautier and Pache 2015). It should be noted that the value of political resources accumulated through philanthropy is more beneficial for firms that are not government-owned or politically well-connected because “gaining political resources is more critical for such firms” (Wang and Qian 2011, p. 1159). Therefore, there is a consensus in academia that corporate philanthropy can be strategically aligned with political legitimacy (Jia and Wang 2013; Kim 2017) or can be used to respond to government pressure (Kim 2017). Following this reasoning, some studies find supporting evidence that politically connected firms, whose political incentive is considered strong, have a greater propensity to engage in corporate philanthropy (Jia and Wang 2013; Li et al. 2015; Kim 2017; Hao et al. 2020; Yang and Tang 2020). Given the enormous pressure faced by the government during the pandemic, philanthropic donations can be viewed as supporting the government. Thus, firms with the desire to obtain or maintain their political resources (e.g., politically connected firms) should have additional incentives to make COVID-19-related donations.

In contrast, state ownership, another corporate political characteristic, should steer philanthropic decisions in the opposite direction, as the incentive to obtain political resources should be low in state-owned enterprises (SOEs hereafter) (Wang and Qian 2011). Given their inherent connections with the state owner, SOEs may already receive preferential treatment from the government, which dilutes their incentives to use philanthropy to build political ties or respond to the government’s call (Li et al. 2015). Moreover, regulated by the State-owned Assets Supervision and Administration Commission (SASAC), SOEs must follow a rigorous review and approval process to donate, including budgeting and consideration of firm size, profitability, debt burden, and cash flow. Indeed, prior studies provide evidence that SOEs are more reluctant than non-SOEs to donate assets or resources that they control or own (Zhang et al. 2010a, b, c; Gao 2011; Li et al. 2015; Tan and Tang 2016).

Accordingly, in the regression, we interact our main independent variable with the indicators of political connection (*PC*) and state ownership (*SOE*). *PC* is equal to one if the chairman or CEO of an observed firm is a current or former government and military official or a member of the People’s Congress or the People’s Political Consultative Conference. *SOE* indicates whether the ultimate controller is state-owned during the sample period, and zero otherwise. As we can see from Columns (1) and (2) of Panel A in Table 5, the positive coefficients (0.001 and 0.006, with *t*-values of 3.96 and 3.87, respectively) of the interaction between *Confirmed*_*[-3,-1]*_ and *PC* indicate that political connections mitigate the negative philanthropic response to COVID-19. Based on the results in Panel B, we can conclude that politically connected firms are unlikely to follow the trend of conservative philanthropic strategies during difficult times. In contrast, in Columns (3) and (4), the coefficients of *Confirmed*_*[-3,-1]*_ × *SOE* are significant and negative (-0.002 and -0.012, with t-values of -2.26 and -1.76, respectively). Consistent with prior studies, these results show that SOEs are reluctant to make sacrifices to tackle the local COVID-19 crisis.

**Table 5:** Political Motive and Corporate Philanthropic Response to COVID-19

	*(1)*	*(2)*	*(3)*	*(4)*
	*Don_Dum*	*Don_Amt*	*Don_Dum*	*Don_Amt*
*Panel A*				
*Confirmed*_*[−3,−1]*_	− 0.001	− 0.009*	− 0.001***	− 0.006***
	(− 1.33)	(− 1.72)	(− 3.77)	(− 4.01)
***Confirmed***_***[−3,−1]***_** × PC**	0.001***	0.006***		
	**(3.96)**	**(3.87)**		
***Confirmed***_***[−3,−1]***_** × SOE**			− 0.002**	− 0.012*
			**(− 2.26)**	**(− 1.76)**
Constant	0.006***	0.037***	0.007***	0.046***
	(17.56)	(17.63)	(11.00)	(11.12)
*Panel B*				
***Confirmed***_***[−3,−1]+***_ ***Confirmed***_***[−3,−1]***_** × PC**	0.000	− 0.000		
*F-Value*	0.01	0.05		
***Confirmed***_***[−3,−1]+***_ ***Confirmed***_***[−3,−1]***_** × SOE**			− 0.003***	− 0.019***
*F-Value*			**12.85**	**12.22**
Firm fixed effects	Yes	Yes	Yes	Yes
Date fixed effects	Yes	Yes	Yes	Yes
Obs	176,645	176,645	176,645	176,645
Adj. *R*^*2*^	0.011	0.011	0.011	0.011

The *t*-values (in parentheses) are based on heteroskedasticity-consistent standard errors clustered by firm

***, **, and * indicate significance at the 1%, 5%, and 10% levels, respectively (two-tailed)

Variables of interest are marked in bold

### Reputational Motive and Corporate Philanthropic Response to COVID-19

The severe shortage of medical supplies, e.g., face masks, goggles, and antiviral drugs, especially in the early stage of the spread, has attracted huge public attention and put pressure on medical firms. These firms are likely to face huge criticism if they profit from the pandemic (e.g., Boonbandit 2020). Consistent with the strategic view of corporate philanthropy as advertisement (e.g., Navarro 1988; Brown et al. 2006; Lev et al. 2010; Gao et al. 2012; Zhang et al. 2010a, b, c), medical firms may proactively seize this opportunity to present themselves as socially responsible rather than reactively facing public pressure. This reputational consideration should encourage medical firms to become leading responders in donation campaigns during this crisis. In Columns (1) and (2) of Table 6, we interact the main independent variable with *Medical*, a variable indicating whether the observed firm belongs to the medical industry. In Panel A, the interaction coefficients in both columns are significant and positive (0.003 and 0.020, with *t*-values of 3.59 and 3.51, respectively). The results in Panel B further suggest that medical firms are relatively more likely to engage in philanthropic donations when the local spread of the pandemic worsens.

**Table 6:** Reputational Motive and Corporate Philanthropic Response to COVID-19

	*(1)*	*(2)*	*(3)*	*(4)*
	*Don_Dum*	*Don_Amt*	*Don_Dum*	*Don_Amt*
Panel A				
*Confirmed*_*[−3,−1]*_	− 0.001***	− 0.005***	− 0.001***	− 0.006***
	(− 3.12)	(− 3.38)	(− 3.59)	(− 3.96)
***Confirmed***_***[−3,−1]***_** × Medical**	0.003***	0.020***		
	**(3.59)**	**(3.51)**		
***Confirmed***_***[−3,−1]***_** × Direct**			0.001***	0.009***
			**(4.41)**	**(4.65)**
Constant	0.007***	0.044***	0.007***	0.043***
	(10.70)	(10.82)	(10.40)	(10.49)
Panel B				
***Confirmed***_***[−3,−1]+***_ ***Confirmed***_***[−3,−1]***_** × Medical**	0.002*	0.015*		
*F − Value*	**2.74**	**2.71**		
***Confirmed***_***[−3,−1]+***_ ***Confirmed***_***[−3,−1]***_** × Direct**			0.000***	0.003**
*F − Value*			**7.50**	**6.54**
Firm fixed effects	Yes	Yes	Yes	Yes
Date fixed effects	Yes	Yes	Yes	Yes
Obs	176,645	176,645	176,645	176,645
Adj. *R*^*2*^	0.011	0.011	0.011	0.011

The *t*-values (in parentheses) are based on heteroskedasticity-consistent standard errors clustered by firm

***, **, and * indicate significance at the 1%, 5%, and 10% levels, respectively (two-tailed)

Variables of interest are marked in bold

The shortage of non-medical supplies (e.g., food, water, and vegetables) can also generate a similar reputational incentive for firms that sell these products directly to customers. In this area, Shan et al. (2008) investigate corporate donations after the Wenchuan earthquake in China and find that firms selling products directly to customers were more likely to donate than other types of firms. The same measure is also used by Gao et al. (2012). Therefore, in the regression, we include *Direct*, the variable used by Shan et al. (2008), to indicate that the observed firm sells products directly to customers. In Columns (3) and (4), we can see that the coefficients of the interaction between *Confirmed*_*[-3,-1]*_ and *Direct* are also significant and positive (0.001 and 0.009, with *t*-values of 4.41 and 4.65, respectively). Overall, the results in Table 6 confirm that firms with reputational motives will proactively take philanthropic actions to help control the local spread of the pandemic.

### Altruistic Motive and the Destinations of Philanthropy

The fact that during the pandemic, donations were made to the local business environment, Hubei Province, or both, leads us to reflect on the underlying implication of the location of the beneficiaries of philanthropy. Many studies find that the beneficiaries of most philanthropic activities are located in the donor community or near their headquarters (McElroy and Siegfried 1986; Galaskiewicz 1997; Saiia et al. 2003; Muller and Whiteman 2009; Tilcsik and Marquis 2013). A widely accepted explanation is derived from the familiar aphorism “charity begins at home.” In particular, philanthropic giving to the local business environment within which firms must operate is meaningful and can be strategically used by firms (Porter and Kramer 2002; Saiia et al. 2003). More specifically, this “home region bias” in corporate philanthropy can be attributed to firms’ “long standing links in the region” (Muller and Whiteman 2009, p. 593), or alternatively as the importance of “local particularities” (Marquis and Battilana 2009, p. 283) for firms. Therefore, during the COVID-19 outbreak, we expect philanthropic donations with a strategic motive to be mainly targeted at the provinces where the donating firms are located. Conversely, donations from non-Hubei firms to Hubei Province, the most severely affected region, are expected to be more aligned with altruism than pure corporate strategies.

To test this conjecture, in Table 7, we divide the main dependent variable *Don_Dum* into two categories: *Don_Local* and *Don_Hubei. Don_Local* indicates whether the observed firm makes a donation to its headquartered province, and *Don_Hubei* indicates whether the observed firm makes a donation directly to Hubei Province. Firms headquartered in Hubei Province are excluded from this analysis because for these firms, Hubei Province is their local business environment. Next, we include the aforementioned philanthropic determinants in the regression. The interaction coefficients in Column (1) support the idea that pre-existing resource availability (*Healthy* and *HighCash*), political considerations (*PC* and *SOE*), and reputational motives (*Direct* and *Medical*) are closely aligned with local philanthropic choices. In comparison, the results in Column (2) indicate that firms that donate to Hubei Province are relatively less likely to carefully weigh the costs and strategic benefits associated with their donations.

**Table 7:** Altruistic Motive and the Destinations of Philanthropy

	(1)	(2)
	*Don_Local*	*Don_Hubei*
***Confirmed[− 3, − 1]***	− 0.001*	− 0.001***
	**(− 1.67)**	**(− 3.62)**
***Confirmed[− 3, − 1] × Healthy***	0.000*	0.000
	**(1.75)**	(1.32)
***Confirmed[− 3, − 1 × Highcash***	0.001**	0.001**
	**(2.56)**	**(2.35)**
***Confirmed[− 3, − 1] × PC***	0.001**	0.000*
	**(2.48)**	**(1.86)**
***Confirmed[− 3, − 1] × SOE***	− 0.001***	− 0.000
	**(− 3.41)**	(− 1.12)
***Confirmed[− 3, − 1] × Direct***	0.001**	0.000
	**(2.31)**	(1.07)
***Confirmed[− 3, − 1] × Medical***	0.002**	0.001*
	**(2.47)**	**(1.69)**
*Constant*	0.004***	0.003***
	(5.89)	(6.43)
Date fixed effects	Yes	Yes
Firm fixed effects	Yes	Yes
Obs	117,012	117,012
Adj. *R*^*2*^	0.005	0.008

The *t*-values (in parentheses) are based on heteroskedasticity-consistent standard errors clustered by firm

***, **, and * indicate significance at the 1%, 5%, and 10% levels, respectively (two-tailed)

Variables of interest are marked in bold

Based on the above conclusions, in the eyes of shareholders, donations made near the firm’s headquarters are more likely to be rational and should generate post-disaster benefits, while donations to Hubei Province are more likely to be altruistic and seen as a misuse of corporate resources. Accordingly, we also investigate investor perceptions towards the different destinations of philanthropy. Table 8 shows the results. In Column (1), we can see that the 3-day average abnormal return following local donations is  − 0.007, while the average return following donations to Hubei Province decreases to  − 0.022. We further compare these two coefficients and find that the difference (0.015) is significant at the 5% level. Following the test in Table 3, we also include the interactions between these two variables and *Confirm*_*[-3,-1]*_ separately in Column (2) of Table 8. The coefficients of the two interactions and the difference between the two coefficients (0.01, with an F-value of 4.48) indicate that when the local situation worsens, shareholders seem indifferent to local COVID-19-related donations, but are unhappy with sending help outside of the local business environment. Overall, the results in Tables 7 and 8 provide preliminary evidence of the relationship between altruism and the destination of philanthropy.

**Table 8:** Investor Reactions to Philanthropy in Different Destinations

	(1)	(2)
*Dep. Var.* =	*CAR[0,* + *2]*	*CAR[0,* + *2]*
***Don_Local***	− 0.007*	− 0.004
	**(− 1.78)**	(− 0.59)
***Don_Hubei***	− 0.022***	0.001
	**(− 3.86)**	(0.08)
*Don_Local* × *Confirm*_*[−3,−1]*_		− 0.001
		(− 0.48)
***Don_Hubei × Confirm***_***[−3,−1]***_		− 0.011***
		**(− 2.91)**
***Confirm***_***[−3,−1]***_	− 0.002***	− 0.001***
	**(− 5.62)**	**(− 5.60)**
*Constant*	− 0.001*	− 0.001*
	(− 1.66)	(− 1.68)
***Don_Local − Don_Hubei***	0.015**	
*F − Value*	**4.55**	
***Don_Local × Confirm***_***[−3,−1]***_*** − Don_ Hubei × Confirm***_***[−3,−1]***_		0.010**
*F − Value*		**4.48**
Firm fixed effects	Yes	Yes
Date fixed effects	Yes	Yes
Obs	100,608	100,608
Adj. *R*^*2*^	0.093	0.093

The *t*-values (in parentheses) are based on heteroskedasticity-consistent standard errors clustered by firm

^***^, **, and * indicate significance at the 1%, 5%, and 10% levels, respectively (two-tailed)

Variables of interest are marked in bold

### Robustness Tests

#### Tests for Parallel Trends

As mentioned earlier, our fixed effects model is essentially a DID model. However, the most critical assumption in a DID model is the parallel trends assumption. Therefore, in the main test, we include indicators for the number of daily confirmed cases on date *t* and after. For example, *Confirmed*_*[0]*_ (*Confirmed*_*[1,3]*_) represents the natural logarithm of one plus the total number of confirmed cases on date *t* (from *t* + 1 to *t* + 3) in provinces where the observed firms are headquartered. *Confirmed*_*[0]*_ is considered future information, as the daily increase in the number of COVID-19 cases is made public every night. Conceptually, there should be no pre-emptive corporate response to future confirmed cases. The results are provided in Table 9. We can see that the coefficients of all indicators for future confirmed cases are not significant, confirming the idea that our results are more than just associations.

**Table 9:** Results for Parallel Trends Assumption

	*(1)*	*(2)*	*(3)*	*(4)*
	*Don_Dum*	*Don_Amt*	*Don_Dum*	*Don_Amt*
***Confirmed***_***[−3,−1]***_	− 0.000*	− 0.003*	− 0.001**	− 0.004**
	**(− 1.69)**	**(− 1.80)**	**(− 2.15)**	**(− 2.37)**
*Confirmed*_*[0]*_	− 0.000	− 0.002	− 0.000	− 0.002
	(− 0.96)	(− 0.88)	(− 1.14)	(− 1.04)
*Confirmed*_*[1,3]*_	− 0.000	− 0.002		
	(− 0.57)	(− 0.92)		
*Confirmed*_*[1]*_			0.000	− 0.000
			(0.03)	(− 0.00)
*Confirmed*_*[2]*_			0.000	0.002
			(0.93)	(0.92)
*Confirmed*_*[3]*_			− 0.000	− 0.002
			(− 0.59)	(− 0.82)
Constant	0.007***	0.048***	0.007***	0.046***
	(8.94)	(9.27)	(8.47)	(8.75)
Firm fixed effects	Yes	Yes	Yes	Yes
Date fixed effects	Yes	Yes	Yes	Yes
Obs	176,645	176,645	176,645	176,645
Adj. *R*^*2*^	0.010	0.011	0.010	0.011

The *t*-values (in parentheses) are based on heteroscedasticity-consistent standard errors clustered by firm

***, **, and * indicate significance at the 1%, 5%, and 10% levels, respectively (two-tailed)

Variables of interest are marked in bold

### Controlling the Effects of Prior Donations and Peer Pressure

There are some confounding factors that need to be considered in our study, such as the peer effect documented in prior philanthropic studies (e.g., Reyniers and Bhalla 2013). It is also likely that a firm will only donate once for a particular event. To mitigate these concerns, we construct two variables, *Peer_Don* and *Prior_Don*, as proxies for peer pressure and prior donations, respectively. Specifically, *Peer_Don* is a variable indicating whether any firm in the same industry made COVID-19-related donations before *t-3*. *Prior_Don* indicates whether the observed firm made COVID-19-related donations before *t-3*. The results are shown in Table 10. Based on the negative coefficients of *Prior_Don*, firms are unlikely to make consecutive donations over a short period. The positive coefficients of *Peer_Don* support the idea of imitation in philanthropic behavior during the pandemic. However, the coefficients of the main independent variable, *Confirmed[-3,-1]*, remain negative. Overall, these confounding factors do not contradict our main results.

**Table 10:** Controlling the Effects of Prior Donations and Peer Pressure

	(1)	(2)	(3)	(4)	(5)	(6)
	Don_Dum	Don_Dum	Don_Dum	Don_Amt	Don_Amt	Don_Amt
**Confirmed**_**[−3,−1]**_	− 0.001*	− 0.001***	− 0.001***	− 0.005***	− 0.004***	− 0.005***
	**(− 1.99)**	**(− 2.59)**	**(− 2.61)**	**(− 2.88)**	**(− 2.87)**	**(− 2.89)**
***Prior_Don***	− 0.011***		− 0.012***	− 0.077***		− 0.078***
	**(− 3.72)**		**(− 3.68)**	**(− 3.86)**		**(− 3.93)**
***Peer_Don***		0.008***	0.008***		0.055***	0.056***
		**(5.92)**	**(5.98)**		**(6.17)**	**(6.23)**
Constant	0.007***	− 0.001	− 0.000	0.046***	− 0.006	− 0.005
	(8.38)	(− 0.41)	(− 0.35)	(10.99)	(− 0.66)	(− 0.60)
Firm fixed effects	Yes	Yes	Yes	Yes	Yes	Yes
Date fixed effects	Yes	Yes	Yes	Yes	Yes	Yes
Obs	176,645	176,645	176,645	176,645	176,645	176,645
Adj. *R*^*2*^	0.011	0.011	0.011	0.011	0.011	0.012

The *t*-values (in parentheses) are based on heteroskedasticity-consistent standard errors clustered by firm

***, **, and * indicate significance at the 1%, 5%, and 10% levels, respectively (two-tailed)

Variables of interest are marked in bold

### Corporate Philanthropic Response Over Time

As the process of most corporate decisions normally takes well over three days, it is doubtful whether the choice of a 3-day window for the main independent variable, *Confirmed*_*[-3,-1]*_, can fully capture firms’ responses. Although it is impossible to know the exact number of days required for each philanthropic decision during the pandemic, a short window can only go against our results, since our focus is on the immediate corporate response to recent information on COVID-19. Moreover, because the economic uncertainty induced by adverse events tends to decrease over time (Muller and Kräussl 2011b), firms’ responses to previous information should be muted over time. Having said that, in Table 11, we use alternative windows, varying from 1 to 30 days, to measure the independent variables. The definitions of these independent variables are similar to *Confirmed*_*[-3,-1]*_. For instance, *Confirmed*_*[-5,-1]*_ is the natural logarithm of one plus the total number of confirmed cases between *t*-1 and *t*-5 in the province of the headquarters of the observed firm. The coefficients of *Confirmed*_*[-1]*_ are negative but not significant, which may be due to the time required for firms to respond. But apart from this, there is an apparent downward trend in both the coefficients and R-squared values as the window expands.^23^ When the lag period is more than five days, the associations between donation likelihood (donation amount) and the number of recent local COVID-19 cases become insignificant. This pattern suggests that firms place relatively more weight on more recent information when making philanthropic decisions. Overall, we consider that the choice of window is appropriate for our study.

**Table 11:** Corporate Philanthropic Response Over Time

	(1)	(2)	(3)	(4)	(5)	(6)	(7)	(8)	(9)
Panel A: Using the Independent Variables in Different Windows with *Don_Dum*									
* Dep. Var.* =	*Don_Dum*								
* Confirmed*_*[-1]*_	− 0.000								
	(− 1.13)								
*** Confirmed***_***[−2,−1]***_		− 0.001**							
		**(− 2.12)**							
*** Confirmed***_***[−3,−1]***_			− 0.000**						
			**(− 2.06)**						
*** Confirmed***_***[−4,−1]***_				− 0.000**					
				**(− 2.09)**					
* Confirmed*_*[−5,−1]*_					− 0.000				
					(− 1.46)				
* Confirmed*_*[−7,−1]*_						− 0.000			
						(− 0.04)			
* Confirmed*_*[−10,−1]*_							0.000		
							(0.09)		
* Confirmed*_*[−20,−1]*_								0.000	
								(0.37)	
* Confirmed*_*[−30,−1]*_									− 0.000
									(− 0.30)
Constant	0.006***	0.007***	0.007***	0.006***	0.006***	0.004***	0.003***	0.002*	0.002
	(12.04)	(10.47)	(10.03)	(9.44)	(8.16)	(5.72)	(4.27)	(1.72)	(0.58)
Firm fixed effects	Yes	Yes	Yes	Yes	Yes	Yes	Yes	Yes	Yes
Date fixed effects	Yes	Yes	Yes	Yes	Yes	Yes	Yes	Yes	Yes
Obs	173,040	169,435	165,830	162,225	158,620	151,410	140,595	104,545	68,495
Adj. *R*^*2*^	0.010	0.010	0.010	0.010	0.010	0.008	0.007	0.005	0.004

	(1)	(2)	(3)	(4)	(5)	(6)	(7)	(8)	(9)
Panel B: Using the Independent Variables in Different Windows with *Don_Amt*									
* Dep. Var.* =	*Don_Amt*								
* Confirmed*_*[−1]*_	− 0.002								
	(− 1.43)								
*** Confirmed***_***[−2,−1]***_		− 0.004**							
		**(− 2.45)**							
*** Confirmed***_***[−3,−1]***_			− 0.004**						
			**(− 2.33)**						
*** Confirmed***_***[−4,−1]***_				− 0.003**					
				**(− 2.33)**					
*** Confirmed***_***[−5,−1]***_					− 0.002*				
					**(− 1.69)**				
* Confirmed*_*[−7,−1]*_						− 0.000			
						(− 0.23)			
* Confirmed*_*[−10,−1]*_							− 0.000		
							(− 0.02)		
* Confirmed*_*[−20,−1]*_								0.000	
								(0.21)	
* Confirmed*_*[−30,−1]*_									− 0.001
									(− 0.30)
Constant	0.037***	0.043***	0.042***	0.041***	0.036***	0.025***	0.021***	0.011*	0.014
	(12.24)	(10.69)	(10.19)	(9.68)	(8.34)	(5.82)	(4.32)	(1.82)	(0.58)
Firm fixed effects	Yes	Yes	Yes	Yes	Yes	Yes	Yes	Yes	Yes
Date fixed effects	Yes	Yes	Yes	Yes	Yes	Yes	Yes	Yes	Yes
Obs	173,040	169,435	165,830	162,225	158,620	151,410	140,595	104,545	68,495
Adj. *R*^*2*^	0.010	0.010	0.010	0.010	0.010	0.008	0.007	0.006	0.004

The *t*-values (in parentheses) are based on heteroskedasticity-consistent standard errors clustered by firm

***, **, and * indicate significance at the 1%, 5%, and 10% levels, respectively (two-tailed)

Variables of interest are marked in bold

### Alternative Independent Variable and Sample

The publicly disclosed COVID-19 data include not only confirmed cases, but also cases of recovery and death. As it seems unlikely that an increase in the number of recovery cases will trigger donations, we also use the daily number of deaths to construct our independent variable. Therefore, in Panel A of Table 12, we replace *Confirmed*_*[-3,-1]*_ with *Death*_*[-3,-1]*_ and find consistent results (the coefficients are  − 0.001 and  − 0.007, respectively; *t*-values are  − 1.82 and  − 1.91, respectively). Our interpretation of the decreased significance level is that on most media platforms, the number of confirmed cases comes first to the public and is therefore likely to attract more attention than the number of deaths.

**Table 12:** Alternative Independent Variable and Sample

	*(1)*	*(2)*
	*Don_Dum*	*Don_Amt*
Panel A: Replacing *Confirmed*_*[−3,−1]*_ with *Death[− 3,1]*		
*** Death***_***[−3,−1]***_	− 0.001*	− 0.007*
	**(− 1.82)**	**(− 1.91)**
Constant	0.006***	0.035***
	(28.87)	(28.25)
Firm fixed effects	Yes	Yes
Date fixed effects	Yes	Yes
Obs	176,645	176,645
Adj. *R*^*2*^	0.010	0.011

	*(1)*	*(2)*	*(3)*	*(4)*
	*Excluding Hubei Province*		*Excluding Wuhan City*	
	*Don_Dum*	*Don_Amt*	*Don_Dum*	*Don_Amt*
**Panel B:** Excluding Firms Headquartered in Hubei Province/Wuhan City				
*** Confirmed***_***[−3,−1]***_	− 0.000*	− 0.003**	− 0.001**	− 0.004**
	**(− 1.65)**	**(− 1.97)**	**(− 2.12)**	**(− 2.37)**
Constant	0.006***	0.040***	0.007***	0.042***
	(10.49)	(10.66)	(10.57)	(10.77)
Firm fixed effects	Yes	Yes	Yes	Yes
Date fixed effects	Yes	Yes	Yes	Yes
Obs	172,039	172,039	173,754	173,754
Adj. *R*^*2*^	0.010	0.011	0.010	0.011

	*(1)*	*(2)*
**Panel C:** Results Using a Cross-sectional Sample		
	*Don_Dum*	*Don_Amt*
*** Confirm***	− 0.020***	− 0.048***
	**(− 3.19)**	**(− 2.91)**
* Size*	0.033***	0.098***
	(6.11)	(6.82)
* Lev*	− 0.004	− 0.018
	(− 0.13)	(− 0.24)
* Cash*	0.046	0.082
	(0.80)	(0.58)
* ROA*	0.004**	0.010***
	(2.40)	(2.58)
* Growth*	0.000**	0.001**
	(2.48)	(2.24)
* Age*	0.002**	0.006**
	(2.13)	(2.56)
* SOE*	− 0.050***	− 0.134***
	(− 3.52)	(− 3.65)
Constant	− 0.059	3.409***
	(− 1.02)	(22.34)
Industry fixed effects	Yes	Yes
Province fixed effects	Yes	Yes
Obs	3,496	3,496
Adj. *R*^*2*^	0.049	0.056

The t-values (in parentheses) are based on heteroskedasticity-consistent standard errors clustered by firm

^***^, **, and * indicate significance at the 1%, 5%, and 10% levels, respectively (two-tailed)

Variables of interest are marked in bold

Another concern is that the negative response to COVID-19 may only exist in the most affected areas. For example, it is possible that only firms in Hubei Province are reluctant to make local donations. Our main response to this concern is that firm fixed effects should perfectly control for any regional pattern. However, to further address this concern, we exclude firms headquartered in either Hubei Province or Wuhan city and rerun the main test. As shown in Panel B of Table 12, the results remain consistent. Therefore, the documented negative response to COVID-19 is not just a regional phenomenon in Hubei Province or Wuhan city.

As the adverse events investigated in prior related studies are one-time events, most of these studies use a cross-sectional sample. To show the consistency of our results with these studies, in Panel C of Table 12, we restructure our panel data into a cross-sectional sample and rerun the regression. Here, *Don_Dum* (*Don_Amt*) represents the likelihood (the total amount) of donations during the sample period, and *Confirm* is calculated as the natural logarithm of the total number of confirmed cases in the province where each firm is headquartered during the sample period. We also include industry and province fixed effects in the regression. Overall, consistent with the main results, firms in the most affected areas are reluctant to make donations during the pandemic.

## Conclusion

In this study, we examine corporate philanthropic decisions in response to the local economic uncertainty induced by the COVID-19 crisis. From the strategic perspective, when COVID-19 spreads locally, firms may adopt proactive strategies, including philanthropic donations, to limit its spread and mitigate the induced economic uncertainty, since firms cannot thrive in a degraded environment. However, from the perspective of non-trivial costs, increased economic uncertainty should force firms to take a close look at their resource allocation decisions through the economic lens. During this difficult time, firms may be more concerned about their survival, and thus adopt cost containment strategies and reduce their spending on philanthropy.

Using data on corporate donations related to COVID-19 in China, we find that at the provincial level, the likelihood and amount of COVID-19-related donations decrease with the number of local COVID-19 cases, implying a retrenchment of philanthropic giving in the COVID-19 crisis. The results also show that investors’ short-term reactions to COVID-19-related donations are negative, especially when the local spread of the virus is severe. Following the proverb “prosperity makes friends, adversity tries them,” the above results suggest that firms and investors are unlikely to take socially responsible action amid the COVID-19 crisis. Further analyses show that the negative response at the provincial level is modified by firm-level determinants of corporate philanthropy, including pre-existing resource availability, political and reputational motives. In particular, firms with abundant resources are actually more likely to make donations in response to the local pandemic spread. Politically connected firms, whose incentive to gain political resources is considered strong, also tend to react less negatively to the local spread of the virus than non-connected firms. In contrast, due to the inherent connection with the state, the philanthropic response of SOEs is more negative than that of non-SOEs. Firms with products falling short of demand, such as those in the medical industry and those selling products directly to customers, have a greater propensity to make donations than other types of firms. These results imply that firms perform cost–benefit analyses when deciding whether or not to help solve social problems. Moreover, we find preliminary evidence that compared with donations made to Hubei Province, donations in the local business environment are more aligned with the above firm-level determinants. This alignment echoes the argument made in prior studies that strategic philanthropy generally focuses on the local business environment (e.g., Porter and Kramer 2002; Saiia et al. 2003). Overall, our multilevel results present a comprehensive picture of corporate philanthropic decisions amid the COVID-19 crisis.

Our study contributes to the literature in the following ways. First, our study extends the institutional analysis of corporate philanthropy in the literature. The majority of prior related studies assume that the features of the local environment are stable over time, and focus specifically on the role of government intervention in the context of developing countries. In response to the criticism of the ignorance of “institutional dynamics” (Gautier and Pache 2015, p. 362) in philanthropic studies, we investigate how firms, as a group at the provincial level, respond to the time-series change of economic uncertainty in the local business environment. Besides, our findings using the Chinese setting respond to the call for more research on philanthropy in developing countries (Gautier and Pache 2015, p. 362). While prior studies support that firms tend to use disaster-related philanthropy to seek legitimacy in these countries, our findings provide evidence against this stereotype and suggest that even in developing countries, the influence of institutional pressure on corporate decisions is largely mitigated by economic concerns in times of crisis.

Second, our study responds to the call for multilevel analysis of corporate philanthropy (Liket and Simaens 2015, p. 302). Specifically, our results show that the economic concerns induced by COVID-19 in the local environment can be mitigated or even outweighed by factors at the organizational level. This interaction between factors at the institutional and organizational levels suggests that each firm will perform its own cost–benefit calculation for its philanthropic decisions. From this aspect, our study offers preliminary evidence on the underexplored decision-making processes involved in corporate philanthropy (Gautier and Pache 2015, p. 363).

Third, our study complements prior studies on corporate philanthropic response to disasters (e.g., Crampton and Patten 2008; Patten 2008; Zhang et al. 2010a, b, c; Zhang et al. 2010a, b, c; Gao 2011; Muller and Kräussl 2011a, b; Gao et al. 2012; Li et al. 2015; Muller and Whiteman 2016). To the best of our knowledge, this is the first study to on the immediate response to adverse events. Our results show that not only firms but also investors are very sensitive to the constant change in economic uncertainty in their local business environment during times of crisis.

One of the methodological shortcomings shared by most studies on philanthropic responses is the choice of cross-sectional data, mainly because the adverse events in these studies (e.g., hurricanes, earthquakes) are relatively isolated in time and space. Because these studies cannot control for unobservable factors using fixed effects in cross-sectional data, most have tried to avoid overinterpretation and preferred to use the word “association.” In our study, as mentioned earlier, the construction of the daily panel sample, along with the application of the fixed effects model, can largely mitigate this endogeneity concern.

### Limitations and Further Research

We acknowledge that there are several limitations in our study, which should be addressed in future research. One major limitation is that we adopt a strict definition of corporate philanthropy and do not consider other types of corporate social activities. The negative philanthropic response to COVID-19 at the provincial level does not signal the “death” of other corporate social behaviors in difficult times. Compared with donations, which are a direct outflow of corporate resources, volunteer activities are relatively less expensive and may be strategically aligned with the core operations of firms. For example, ZTE Corporation has actively protected the health of its employees and helped operators build and secure 5G networks in Wuhan (ZTE 2020). This type of action falls within the definition of strategic philanthropy and is likely to increase after reopening. Future studies could focus on the broad CSR picture and investigate whether, at different stages of the COVID-19 crisis, there are systematic changes in the different types of corporate social responses. The policy implications of this type of research can help authorities determine whether and to what extent they can rely on CSR activities to solve social problems.

We also do not address the question whether the negative philanthropic response at the provincial level is irrational or not. In the work of Dessaint and Matray (2017), the increase in cash holdings by firms located in the vicinity of the hurricane-stricken area is interpreted as managers’ overreactions to the economic consequences of hurricanes. In terms of both the novelty of the crisis and media coverage, COVID-19 should cause more salient risks than hurricanes and is thus more likely to trigger overreactions. Consequently, the negative bias in philanthropy during COVID-19 may also reflect managers’ fear of the pandemic. One possible way to exclude the behavioral interpretation of our results is to follow Dessaint and Matray (2017) and investigate whether the negative philanthropic response is temporary. But even so, as mentioned in the third robustness test, the temporary response can still be explained from a rational perspective, as the economic uncertainty associated with disasters tends to decrease over time (Muller and Kräussl 2011b). Alternatively, future research could examine the outcomes of philanthropy (Liket and Simaens 2015). If the results confirm that COVID-19-related donations generate post-crisis benefits and that firm survival is unlikely to be an issue, then negative philanthropic bias is more likely to be the result of fear than rational decisions.

Another overlooked aspect concerns the communication strategies used in philanthropy. In our sample, some firms chose to keep a low profile and even did not make an official donation announcement, while donations made by others were repeatedly covered on different media platforms. The causes and consequences of this phenomenon are promising avenues for future research.

Finally, future research could further explore how cross-national variations in institutional features affect philanthropic responses to the pandemic. A very notable trend is the debate on the different reactions of authoritarian and democratic countries (e.g., Kleinfeld 2020). The Chinese government has mobilized the entire nation to deal with COVID-19 and has strictly implemented a nationwide strategy. In contrast, many democratic countries (or regions) have managed to control the virus without lockdown, for example, South Korea, Taiwan, and Hong Kong (McKinsey 2020). In the US, each state has developed its own policies in response to the pandemic rather than following a unified strategy (Lee et al. 2020). As the induced economic uncertainty is strongly associated with COVID-19-related policies, different government responses should lead to different corporate decisions.

## Figures and Tables

**Figure:**
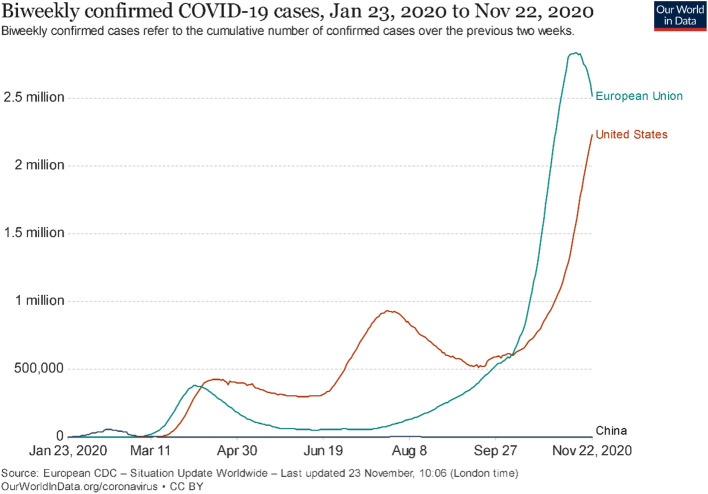
Source: directly from COVID-19 dataset, Our World in Data.

## Data Availability

The COIVD-19 data that support the results of this study are from Tencent’s search engine at: https://news.qq.com/zt2020/page/feiyan.htm. Corporate financial data are obtained from the China Stock Market & Accounting Research (CSMAR) Database.

## Appendix A: Examples of news on COVID-19-related donations

Example 1: Donations made by the GAC Group.

“Cumulative Donation of over RMB22 million! GAC Group Donated RMB8.35 Million in Cash, Vehicles and Protective Equipment to Guangzhou Municipal Health Commission.

February 5, 2020.

In the afternoon of February 4, 2020, the Guangzhou Municipal Health Commission held the “Guangzhou COVID-19 Prevention and Control Medical Materials Donation Ceremony.” GAC Group donated RMB5 million cash and 20 GAC Motor vehicles worthy of RMB 3 million as well as protective equipment such as 2000 surgical gowns, 10,000 N95 masks to the Guangzhou Municipal Health Commission through the Guangzhou Charity Association. The total donation, valued at RMB 8.35 million, would be distributed to various hospitals by the Guangzhou Municipal Health Commission.

Since January 26, in order to support COVID-19 prevention and control, GAC Group and its affiliates have made a number of donations. On January 26, it announced that it will donate RMB 5 million in cash and materials together with GAC Honda, GAC Toyota and GAC Motor. On January 31, an additional donation of RMB 9 million in cash and materials was announced. Together with the new donation today, the accumulated donation amount of GAC Group has exceeded RMB 22 million, and GAC Group alone made a contribution of over RMB 15 million.”

*Source:* https://www.gac.com.cn/gw_en/xwzx_en/qydt_en/20200205/detail-18002.shtml

Example 2: Donations made by IReader Technology Co.

“IReader Technology Co.: RMB2 million donated to two Hubei hospitals.

IT Home News, January 30. According to iReader’s official Weibo account, the company donated 1 million yuan each to Renmin Hospital of Wuhan University and Zhongnan Hospital of Wuhan University, or a total of 2 million yuan, for the prevention and control work in Hubei Province, to win the fight against the epidemic …” This news article also provides the digital cash transfer certificate related to the donation. We can see from the certificate that the actual donation date is January 29, 2020.

*Note*: This news article has been translated from Chinese to English.

*Source:* https://www.ithome.com/0/470/583.htm

## Appendix C: Variable Descriptions

**Table:**

*Variable*	*Definition*
*Don_Dum*	*A dummy variable indicating whether the observed firm makes a donation on date t*
*Don_Amt*	*Donation amount, calculated as the natural logarithm of one plus the value of cash and supplies (in RMB10,000) donated by the observed firm on date t*
*Confirmed*_*[-3,-1]*_	*The natural logarithm of one plus the total number of confirmed cases between t-1 and t-3 in the province where the observed firm is headquartered*
*Death*_*[-3,-1]*_	*The natural logarithm of one plus the total number of deaths between t-1 and t-3 in the province where the observed firm is headquartered*
*Healthy*	*An indicator of financial health, which takes the value of one if the Z*_*China*_* score of the observed firm is in the top decile, and zero otherwise*
*HighCash*	*An indicator of corporate cash holdings, which takes the value of one if the cash holding of the observed firm is in the top decile, and zero otherwise*
*Medical*	*An indicator that takes the value of one if the observed firm belongs to the medical industry, and zero otherwise*
*Direct*	*An indicator that takes the value of one if the product of the observed firm is directly sold to customers*
*PC*	*An indicator of political connections, which takes the value of one if the firm’s chairman or CEO is a current or former government and military official or a member of the People’s Congress or the People’s Political Consultative Conference, and zero otherwise*
*SOE*	*An indicator of state-owned enterprises, which takes the value of one if the ultimate controller is the state at the start of the sampling period, and zero otherwise*
*Don_Local*	*A dummy variable indicating whether the observed firm makes a donation to the province of its headquarters on date t*
*Don_Hubei*	*A dummy variable indicating whether the observed firm makes a donation to Hubei Province on date t*
*Size*	*Firm size, calculated as the natural logarithm of total assets*
*Lev*	*Leverage, calculated as total liabilities divided by total assets*
*Cash*	*Cash holdings, calculated as total cash divided by total assets*
*ROA*	*Profitability, equal to net income divided by total assets*
*Growth*	*Sales growth, calculated as the change in sales from the last year to the current year, divided by sales in the last year*
*Age*	*Firm age, calculated as the number of years the observed firm has been listed on the A-share market*
*Peer_Don*	*A variable indicating whether all firms in the same industry made COVID-19-related donations before t-3*
*Prior_Don*	*A variable indicating whether the observed firm made COVID-19-related donations before t-3*

## Footnotes

1 For example, on January 28, 2020, Shenzhen Neptunus Bioengineering Co., Ltd, a Chinese listed firm, announced that it would proactively participate in the prevention and control of the pandemic by working overtime to provide medical supplies (Li, 2020).

2 It should be noted that institutional theory also suggests that firms have incentives to make social efforts to protect the local business environment. According to this theory, each organization tries to seek or maintain its legitimacy with institutional constituents to compete for scarce resources (Scott, 1995; Deephouse, 1999). Legitimacy can be accumulated through actions of an entity that “are desirable, proper, or appropriate within some socially constructed system of norms, values, beliefs, and definitions” (Suchman, 1995, p. 574). Hence, the legitimacy concern can motivate firms to take socially desirable actions in their business environment. Obviously, philanthropic donations to help fight COVID-19 fall into this category. This incentive is especially strong in the Chinese setting, where the government wields enormous regulatory power and controls crucial business resources (e.g., Zhang, Rezaee, and Zhu, 2010a, b, c; Wang and Qian, 2011; Zhao, 2012). Moreover, if one firm can gain legitimacy through such actions, others will develop a similar strategy, because not to do so will adversely influence its competitiveness (e.g., Deephouse, 1999; Marquis et al. 2007). Essentially, the strategic perspective and institutional theory share the idea that firms’ economic goals can be integrated with social goals (e.g., Maas and Liket, 2011). For the sake of parsimony, we limit the discussion on institutional theory in the hypothesis development.

3 According to Liket and Simaens (2015), the institutional level of analysis includes research “without referring to organizational characteristics or managers within the company” (p. 288) and that “concern[s] society, or specific effects of industries or nations” (p. 289). Because firm fixed effects control for time-invariant factors at the organizational level, such as firm size and profitability, the coefficient of the independent variable in Model (1) captures the average impact of COVID-19 on philanthropic donations made by firms in the same province. The study by Liket and Simaens (2015) is further discussed in the literature review.

4 There may be some confusion regarding our institutional level of analysis because, as mentioned in the Literature Review section, the term “institution” in the literature of corporate philanthropy usually refers to country-specific characteristics, such as government intervention and political resources, especially in the context of emerging markets (e.g., Su and He, 2010; Zhang, Rezaee and Zhu, 2010a, b, c; Wang and Qian, 2011; Gao, 2011; Li et al. 2015; Tan and Tang, 2016; Hao et al. 2020; Yang and Tang, 2020). It is important to note that our institutional analysis investigates how changes in the same local environment affect corporate philanthropic decisions, rather than how the unique time-invariant features of the Chinese context shape corporate philanthropic practices. In the Further Analyses section, we also examine two institutional characteristics shared by many Chinese firms: political connections and state ownership.

5 More precisely, proxies for reputational motives vary by industry and should therefore be classified as meso-level indicators. For brevity, we ignore the differences between industry-level and firm-level analyses.

6 This information is obtained from the WHO’s website related to COVID-19 (https://covid19.who.int/).

7 Specifically, based on a news article published on January 25, 2020 by ThePaper.cn, 19 hospitals in Wuhan City made official emergency requests to the public and provided a detailed list of medical supplies in shortage. For more details, please see the following website: https://www.thepaper.cn/newsDetail_forward_5632160

8 This leads to a 2 × 2 crisis matrix, including conventional crisis (predictable and influenceable), unexpected crisis (unpredictable but influenceable), intractable crisis (predictable but uncontrollable), and fundamental crisis (unpredictable and uncontrollable).

9 In the US, different states have adopted different responses to the pandemic (Lee et al. 2020). Therefore, the economic implications for the same number of new confirmed cases are likely to be different across states.

10 Small-scale disasters are defined as disasters with damages of less than US$1 billion, while major disasters are identified as the top 25% of US$1 billion disasters.

11 According to the white paper titled “Fighting COVID-19: China in Action” issued by China’s State Council Information Office, “local authorities and government departments were required to take measures matching the corresponding levels of emergency response and ensure an orderly return to work and normal life.” For example, from January 23 to January 29, 2020, all provinces in mainland China activated a Level-1 public health emergency response.

12 These indicators include implied stock market volatility, newspaper-based economic policy uncertainty, Twitter chatter about economic uncertainty, subjective uncertainty about future business growth, and disagreement between professional forecasters on future GDP growth.

13 Firms listed after 2018 are excluded because public information about these firms, such as annual financial reports and ownership structure, was not available during the main outbreak period.

14 Indeed, data at the provincial and city levels have been available since January 22, 2020. To obtain the daily increase in the number of cases, we remove January 22 from our sample period. In addition, the restriction and lockdown policy was gradually implemented after January 23, 2020. As our hypothesis development discusses the economic consequences of the lockdown and restriction policy, it is appropriate to select a sample period starting after the first implementation of this policy. Our results remain consistent when this date is included.

15 We obtain our data from the following website: https://news.qq.com/zt2020/page/feiyan.htm

16 Consistent with Dessaint and Matray (2017), corporate decisions are more affected by events in the headquarters than operating regions, because the decision makers are normally in the headquarters. Thus, the local business environment is identified as a firm’s headquartered province rather than its operating provinces.

17 During the sample period, we search for various keywords, including firm name, donation, and COVID-19, in all corporate disclosures and major search engines. Then, we manually go through each item and determine if the donation is related to COVID-19.

18 In case of duplicate coverage for the same donation, the earliest news report date (or announcement date) is selected.

19 In most cases, the action date was before the news report date (or the announcement date). In these cases, using the action date as the donation date can eliminate the concern that firms may strategically time their disclosure of past donations. Conversely, when firms announced their donations before taking action, we choose the announcement date as the donation date. This decision raises the potential concern that firms may announce their donations but subsequently refuse to take action. However, it should be noted that this type of action is costly. Specifically, according to the Charity Law of the People’s Republic of China: “in breach of the donation agreement, donors fail to deliver donated assets exceeding the time limit, and where one of the following circumstances exists, charitable organizations and other persons accepting the donations can request delivery; where the donors refuse to deliver, charitable organizations and other persons accepting donations can apply to the people’s courts for an order for payment or raise a lawsuit: (1) Where donors have publicly promised to donate through radio, television, press, the Internet, or other such media.” In addition, related studies, such as Muller and Kräussl (2011b), do not distinguish donation announcement from actual actions as well. As a result, potential dishonest donation behavior, which merits future investigation, should not be a big threat to the choice of announcement date.

20 If a firm makes multiple donations in one day, the donations made on that day will be the aggregate amount.

21 Although the dependent variable here is a dummy, a linear fixed effects model can generate unbiased estimates according to many econometrists (e.g., Riedl and Geishecker, 2014). Alternatively, we use a logit fixed effects model, and the coefficient of *Confirmed*_*[-3,-1]*_ remains significant and negative.

22 As our sample period is very short and close to the Spring Festival, it is almost impossible for a firm to have changed its managers, industry, or location during the short sample period, especially as the entire nation was on hiatus due to COVID-19.

23 It should be noted that in Table 11, the reason why the sample size decreases with the length of the window is because there are more missing values for longer windows. For example, the values for *Confirmed*_*[-7,-1]*_ become non-missing only after January 30, 2020. This treatment results in a slight difference between the number of observations in the main test and the number of observations here when *Confirmed*_*[-3,-1]*_ is the independent variable.
